# Primate autoimmune disease models; lost for translation?

**DOI:** 10.1038/cti.2016.82

**Published:** 2016-12-30

**Authors:** Bert A 't Hart

**Affiliations:** 1 Department Immunobiology, Biomedical Primate Research Centre, Rijswijk, The Netherlands; 2 University of Groningen, University Medical Center, department Neuroscience, Groningen, The Netherlands

## Abstract

Replacement, reduction and refinement (the 3R's) are the leading principles in translational research with animals. To be useful a model should also be clinically Relevant (the 4th R). Work in a non-human primate model of multiple sclerosis, the experimental autoimmune encephalomyelitis model, reveals an inherent conflict among these 4R principles. The impossibility to harmonize all 4R's forms a major challenge when the model is applied in preclinical drug development.

## Introduction

Ageing western societies are facing an increasing burden of immune-based disorders (allergy, chronic inflammation, autoimmunity) for which no adequate treatments are available. Much has already been written about the major problems in the translation of new scientific concepts into effective treatments (=forward translation), for which the poor predictive value of clinical success of currently used animal models has been blamed (for example, Kola and Landis^1^ and Arrowsmith and Miller^2^). The vast majority of animal models currently used in the preclinical research of immune-based disorders has been established in a few inbred mouse strains. Although the laboratory mouse has been extremely valuable for our current knowledge of the immune system, its relevance as preclinical model of human disease is debated due to substantial immunological differences with humans. A recent study shows that the immunological distance between those models and humans is not only a reflection of the evolutionary distance, but is also caused by the very clean (specific pathogen-free (SPF)) breeding conditions of the mice, which limits maturation of the immune system.^3^ This translational gap can be bridged by using species which are not only evolutionary closer to humans, but also live under comparable conditions as humans, such as non-human primates (NHP) from captive colonies.^4, 5^

For highly species-specific reagents, such as monoclonal antibodies, which are usually inactive in rodents, transgenic mice expressing the human target molecule or NHP are potentially useful models. However, final preclinical tests of safety and efficacy of a new treatment are often performed in a NHP disease model, which more closely approximates human immunopathology.

The central theme of this perspective is the potential conflict between the high unmet need of translationally relevant preclinical animal models for human autoimmune disease and the necessity to implement the ethical requirements formulated in the 3R principles of animal experimentation in their research.^6^ Underlying this conflict, which threatens the development of effective treatments, is a lack of appreciation that NHP autoimmune disease models can provide unique and translationally relevant information that cannot be obtained from research in lower disease models or the patient.

## Relevance of non-human primate EAE models

The most intensively studied animal model of human autoimmune-mediated inflammatory disease is experimental autoimmune encephalomyelitis (EAE). EAE is not only used as a specific model for the autoimmune neuro-inflammatory disease multiple sclerosis (MS), but also as the most widely studied generic animal model of human autoimmune-mediated inflammatory disease, in which many immunotherapies have been developed. The most commonly used NHP species in EAE research are *Macaca mulatta* (rhesus monkey), *Macaca fascicularis* (cynomolgus monkey) and *Callithrix jacchus* (common marmoset). Although all three species were found to be susceptible to EAE,^7^ the model in marmosets most closely approximates MS in clinical and pathological presentation.^8^ Marmosets are non-endangered small-bodied primates (averaging 300–500 grams at sexually adult age, that is 2 years). The evolutionary distance to man of ±40 million years is reflected by high genetic, immunological and physiological similarity.^9^ The natural habitat is the Amazon forest. Marmosets adapt well to the moderate climate of Western Europe. The Biomedical Primate Research Centre (located in Rijswijk, Netherlands; www.bprc.nl) houses a stable, pedigreed, purpose-bred colony of ±140 marmosets in outdoor enclosures. This outbred colony represents relevant aspects of the human MS population: genetic heterogeneity and the exposure to factors from the environment, which influence the course of autoimmune diseases, such as infections and sunlight (Vitamin D).^10, 11^ Work in marmoset EAE shows that besides infections from environmental pathogens also endogenous viruses, which are engaged in lifelong interaction with the host immune system, such as CMV or EBV,^12^ exert persistent effects on the immune system. The presumed pathogenic role of EBV in MS seems based on the capacity of EBV-infected B cells to recruit strongly pathogenic T cells from the anti-CMV effector memory repertoire.^13^

A unique advantage of the marmoset as experimental disease model is that they are usually born as non-identical twins, which due to sharing of the placental blood stream develop as bone marrow chimeras.^14^ As the immune systems of such non-identical twins mature in the same thymic and bone marrow compartments, they are nevertheless immunologically highly comparable. This enables two-leg experiments in twins, where one sibling receives an experimental treatment and the other a placebo preparation or sham treatment.

The translational relevance of NHP as models of human autoimmune-mediated inflammatory disease lies also in the fact that the primate immune system is modified during the lifelong battle with similar pathogens as the human population is exposed to. This means that a substantial part of the adult immune repertoire has experienced prior antigen challenge and may already be committed to certain functions. This contrasts with the immunological immaturity of the SPF-bred mice strains in which current standard disease models have been established.^3^ The different pathogen experience of mice versus primates, which deepens with aging,^15^ implies for example that strategies aiming at tolerization or reprogramming of pathogenic T cells developed in mice often fail in primates.^16^

The discovery in the marmoset EAE model that the pathogen-educated immune repertoire harbors strongly pathogenic effector memory T cells forms the basis for a novel category of atypical EAE models (see below). These models are induced using antigen-adjuvant formulations lacking danger signals from bacterial factors, which are indispensable for the activation of immature autoreactive T cells in SPF mouse models. It would be highly interesting to test whether SPF mice co-housed with dirty mice^3^ are susceptible to atypical EAE.

## Decreasing public acceptance of animal research

The public support in Western societies for the use of living animals in preclinical research, NHP in particular, is low. The opposition against translational research in living animals conflicts with the high unmet need of safe and effective treatments for chronic inflammatory and degenerative diseases, which form an increasing burden in aging western societies and for which investments in animal research are desperately needed. The negative research climate also makes funding agencies reluctant to financially support exploratory scientific research into the NHP immune system, which is essential for the development of relevant models for preclinical research.

The fastest growing category of promising biotechnological drugs includes the monoclonal antibodies. With these highly specific agents unwanted cells and molecules can be physically or functionally eliminated with high efficacy and usually acceptable side effects.^17^ The high species-specificity of monoclonal antibodies, however, usually precludes safety and efficacy tests in species distant from humans, such as rodents, necessitating the usage of NHP, or for specific questions transgenic mice.^18^ Although relevant information can be obtained from models in transgenic mice it is nevertheless felt that a well-designed study in a relevant NHP disease model provides information on pharmacokinetics/pharmacodynamics, immunogenicity, effective dose levels and potential toxicity (for example, anaphylaxis) that cannot be obtained in lower species. Unfortunately, documented examples of cases where development of a therapeutic mAb was stopped for lack of relevant activity or unexpected toxicity are scarce because such negative studies are usually not published.

Lack of adequate funding is not the only hurdle. Preclinical research with NHP in European Union countries is also increasingly restricted at the political level. On September 22, 2010 the European parliament has adopted directive 2010/63/EU as Europe-wide legislation for the protection of animals used for scientific purposes. The directive, explicitly states that: ‘the use of non-human primates should be permitted only in those biomedical areas essential for the benefit of human beings, for which no other alternative replacement methods are yet available. Their use should be permitted only for basic research, the preservation of the respective non-human primate species or when the work, including xenotransplantation, is carried out in relation to potentially life-threatening conditions in humans or in relation to cases having a substantial impact on a person's day-to-day functioning, i.e., debilitating conditions'. Under this directive a new ethics review system has been installed per November 2015, which has made life for EU-based scientists working with animal disease models substantially more difficult. It is somewhat paradoxical that at the same time the European Commission stimulates translational research into human diseases that should bridge the ‘valley of death' in therapy development. To reach this goal, investments in better animal models, including NHP, will be inevitable.

The fact that directive 2010/63/EU is firmly based on the 3R principles of Replacement, Reduction and Refinement, warrants deeper consideration of the consequences for translational research in NHP autoimmune disease models.

## Ethical challenges

The leading principles in preclinical research with animals have been formulated in the 3R's: Replacement, Reduction and Refinement.^6^ Strict adherence to these principles is now common practice in preclinical research with animals, especially in research with NHP. However, it is rather remarkable that a highly relevant 4th R is lacking, namely the (clinical) Relevance of an animal model. In the following these 4R's will be briefly discussed as well as why the necessity to harmonize them has nade the generation of a clinically more relevant EAE model an acrobatic balancing act.

**Figure 1:**
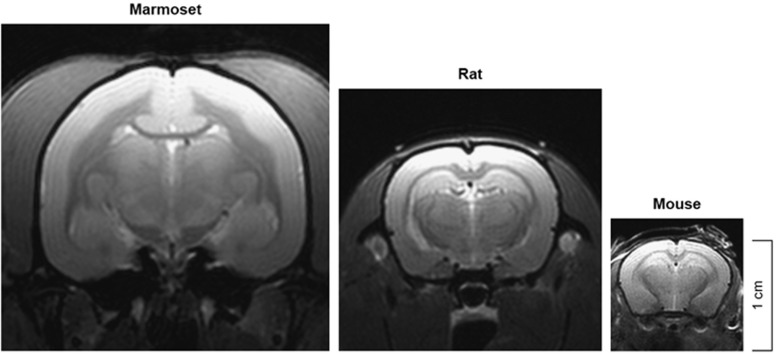
Proton density brain images of a marmoset, rat and mouse. Clearly visible is the higher amount of compact myelin (white matter) in the marmoset brain, which is an important advantage in preclinical studies with the EAE model.

The Replacement principle encourages scientists to use methods in their research that avoid or replace the use of animals. While *in vitro* models with cells or tissues and *in silico* models of human pathology are increasingly used in preclinical research, it is generally felt that the complexity of a pathological process in conjunction with other physiological body systems is only displayed in living animals. In that case the lowest animal species from which relevant information can be obtained should be chosen. This consideration underlies the increasing use of evolutionary distant model systems, such as zebrafish *(Brachydanio rerio*), fruit flies (Drosophila) and C. elegans worms. However, although important insights into novel disease mechanisms can be obtained with these models, it is felt that model systems closer to the patient are needed for the integration of all information into a coherent pathogenic concept that can be translated into new therapies. Especially relevant for MS research is that the architecture of the primate brain is more comparable to the human brain than the brain of rodents (Figure 1).

The Reduction principle encourages scientists to use research methods that provide either comparable levels of information from fewer animals or more information from the same number of animals. The latter was achieved by the application of magnetic resonance imaging techniques,^19^ but compliance with the former condition is more problematic. Project reviewers interpret this parameter usually as the minimal number of animals that should be used for a ‘usable' result. Power calculations are applied to determine the minimal size of experimental and control groups, so that the effect of an experimental variable can be statistically tested. Critical variables in the power calculation are the disease incidence, the anticipated treatment effect and the variation in read-out parameters, such as the time to clinically evident disease or the disease severity. Due to the variable influence of genetic background and environmental factors, ironically being dominant autoimmune disease risk factors, variation is inherent to NHP disease models. The inevitable consequence of variation is that experimental groups may contain clinical low- or non-responders, which reduces disease incidence and thus requires larger group size. In rodent disease models this dilemma is bypassed by using well-established genetically homogeneous (inbred) SPF-bred strains and the usage of potent adjuvants (see below) to obtain high disease incidence and a synchronous disease course. However, a recent study raised questions on the relevance of immunologically immature SPF mice as relevant models of complex human diseases.^3^ Moreover, the usage of strong bacterial adjuvants conflicts with the Refinement and Relevance principles (see below).

The Refinement principle encourages scientists to use methods that minimize the discomfort from experimental procedures to the animals. A major point of concern with respect to the EAE model is that for reproducible disease induction strong adjuvants need to be used to pepper the immunogenic potency of self-antigens by disrupting the regulatory mechanisms that keep autoaggressive T and B cells under control.^20^ The most frequently used is complete Freund's adjuvant (CFA), which is an emulsion of heat-killed mycobacteria (*M. tuberculosis* or *butyricum*) in mineral oil. The mineral oil forms a depot for the finely dispersed antigen solution in aqueous buffer; the mycobacteria provide danger signals needed for ‘awakening' of the tolerized T and B cells. However, CFA is notorious for its seriously detrimental side effects, in particular the induction of severe ulcerative skin lesions at the injection sites, which are clearly caused by the mycobacteria. Usage of CFA in NHP is therefore discouraged.

The 4thR of Relevance: The usage of CFA introduces a mechanistic bias in the EAE model as immune responses against antigens formulated with CFA are skewed towards a pro-inflammatory CD4 dominated profile.^21^ The poor translation record of experimental therapies targeting CD4^+^ T cells from EAE to MS indicates that this cell subset may be less relevant in the human disease than in the animal model.^22^ However, this does not preclude a pathogenic role of CD4^+^ T cells early in the disease process, before the diagnosis has been made.

**Figure 2:**
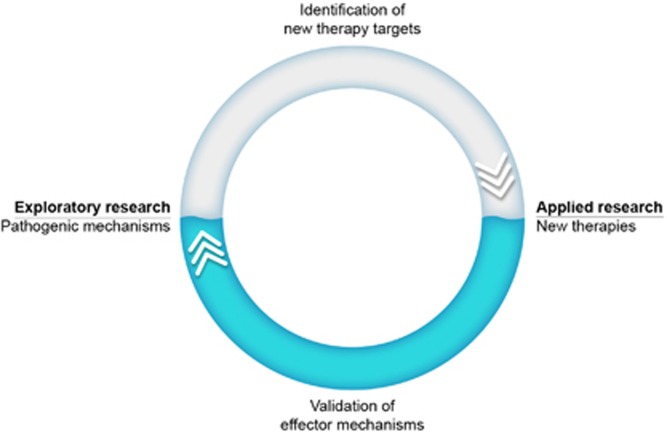
Iterative strategy for refinement of the marmoset EAE model. Exploratory research into new pathogenic mechanisms and applied research into the efficacy of new therapeutics are communicating vessels. When a new therapeutic entity shows similar clinical effects in the EAE model as in MS it can be concluded that the targeted mechanism is clinically relevant. Using this strategy a new pathogenic mechanism involving the interaction of anti-CMV T cells and EBV-infected B cells was discovered.^47^

Compliance of the marmoset EAE model with the 4th R has been achieved via an iterative strategy depicted in Figure 2. In brief, research in the exploratory arm aimed at maximum refinement of the original EAE model, which was induced by immunization with myelin isolated from the brain of an MS patient that was formulated with the bacterial adjuvant CFA.^23^ The stepwise refinement of this highly complex model into the minimally essential components yielded a highly refined model that displayed essential pathological aspects of MS.^8^ In the applied arm the consecutive steps of the refinement process were validated with clinically relevant therapeutic monoclonal antibodies. The in depth characterization of this atypical EAE model is still ongoing, but preliminary data show that the MHC-E restricted CTL that mediate the development of chronic EAE in marmosets^24^ can also be found in MS lesions.^25^

## Marmoset EAE: atypical disease models

As undisputed evidence for an exogenous trigger of MS is lacking, it was proposed that the cause of the disease might be endogenous.^26^ The underlying ‘inside-out' concept implies that autoimmunity in MS essentially represents a (genetically predisposed?) immune hyper-activity against antigens that leak from idiopathic myelin injury.^27^ Small-sized spontaneously appearing injuries in myelin have been detected in the MS brain in the form of microglia nodules^28^ and degenerating oligodendrocytes,^29^ which may actually be two sides of the same coin.^30^ The novel atypical marmoset EAE models revealed presence in the pathogen-educated primate immune system of autoaggressive T cells that are hyper-reactive against the CNS myelin component myelin oligodendrocyte glycoprotein (MOG).^31^

The stepwise dissection of the complex immune reactions raised in marmosets sensitized against human myelin has been reviewed in detail elsewhere.^8^ Here, only the translationally most relevant details are summarized:

- The response of pathogenic T and B cells mediating chronic disease focuses on the quantitatively minor CNS myelin component MOG.
- The uniform initiation and variable evolution of EAE in marmosets sensitized against recombinant human MOG_1–125_ are regulated by distinct pathogenic mechanisms.
- EAE initiation involves the activation of MHC class II/Caja-DRB*W1201-restricted naïve CD4^+^ T cells specific for residues 24–36. This pathway is well-known from rodent EAE models, but the relevance for MS is unclear.^32^
- EAE progression involves the (re-)activation of CD8^+^ effector memory cytotoxic T cells by B cells infected with the EBV-related callithrichine herpesvirus-3. The B cells cross-present the core epitope (residues 40–48) via non-classical MHC class Ib/Caja-E molecules.^33^
- A similar type of cytotoxic T cells has been found in MS lesions in close proximity to HLA-E expressing oligodendrocytes, which seem to be attacked.^25^ In line with this finding we showed that the cytotoxic T cells induce demyelination in the white and grey matter of brain and spinal cord. The EAE model provided evidence that these strongly pathogenic T cells may originate from a repertoire of effector memory T cells elicited by cytomegalovirus, providing a mechanistic explanation for the presumed pathogenic role of this β-herpesvirus in MS.^34^
- The EAE model revealed a crucial pathogenic role of γ1-herpesvirus infection of B cells.^13^ The virus infection seems to induce protection of the proteolysis-sensitive MOG40–48 epitope against destructive processing in B cells and activation of the antigen cross-presentation machinery.^35^

The atypical EAE models in which EAE progression has been modeled, depend on a unique aspect of marmosets, being the crosstalk of auto-aggressive T cells raised against cytomegalovirus with EBV-infected B cells. This seems clinically relevant as both viruses have been implicated as dominant risk factors for MS.^34, 36^

## A prisoner's dilemma

The replacement of CFA for IFA in the translationally relevant atypical EAE models had various important consequences: 1. The discomfort to the animals in experiment was reduced; 2. The immunogenicity of administered biological therapeutics, which limited their activity window, was reduced; 3. The dogma that danger signals are absolutely needed for autoimmunity induction may need to be adjusted; and 4. A new pathogenic mechanism was discovered. Concerning the third issue a caveat may be needed as the injection of antigen/IFA emulsion will certainly cause damage and induce the release of damage-associated molecular patterns, which can relay danger signals to APC through DAMP receptors.^37^ The released DAMPS cause some skin irritation. However, these signals are unable to elicit EAE in genetically susceptible but immunologically immature SPF mice.^38^

The compliance of the primate EAE model with the Refinement and Relevance principles introduced an unforeseen conflict with the Reduction principle. It was observed that the replacement of CFA for IFA produced less robust EAE models, which were more prone to variation in the response against immunization as well as in the response to treatment. A likely explanation is that these refined disease models are more sensitive to the variable influences of genetic and environmental factors. An experiment in which we experienced the consequences was reported recently.^39^ In brief, a powered two-leg study in marmoset twins (*n*=7) immunized with MOG peptide 34–56/IFA tested the efficacy of a mAb raised against the human IL-7 receptor CD127.^39^ One sibling of each twin received the therapeutic mAb and the other a placebo preparation. We observed that one twin pair did not develop clinical EAE within the 150 days observation period, while in the six twin pairs that did develop EAE no statistically significant effect was observed at the group level. However, at the individual twin level we observed that three twins with a fast disease evolution responded to the treatment, while no effect of the mAb was observed in three twins with a slowly evolving disease. The lack of statistical significance at the group level made publication of the data highly problematic.

How should this experiment be interpreted? Should further development of the mAb be stopped because a significant effect of the treatment could not be proven, even not when the number of animals per group is doubled or tripled? Or might the mAb be clinically relevant for a subset of the patients, namely those with fast disease progression? This is not a theoretical issue as also in MS patients variation in the response to treatment (with interferon-β) has been observed.^40^

The prisoner's dilemma here is that when there are no markers for disease progression rate that can be used for pre-selection of high responder animals, the only way to achieve statistical significance is increasing the group size. This creates a conflict with the Reduction principle.

## Ranking the 4R's

There seems to be no easy solution for the apparent conflict between (statistical) significance and (clinical) relevance for the highly refined EAE models. One solution could be that reviewers of grants and publications on proof of concept studies choose not to apply the normal sample size dogmas for homogeneous models, such as those in inbred/SPF mice, to studies in the more complex and heterogeneous disease models in NHP.^41, 42^ The central argument is that the value of a study does not increase proportionally with the number of animals added to a test group, while the burden from discomfort on study participants does increment proportionally.^43^

Another way to approach the problem might be to weigh the potential impact of each R on the study outcome.

1. Relevance: It seems unlikely that relevant results can be obtained from preclinical tests of a novel treatment in an animal model that is irrelevant for the targeted disease. Important in this context is that not only the clinical and pathological end-points of a model should represent the targeted disease in humans, but also the pathogenic mechanisms that lead to those end-points as these are targeted by a treatment. The difficulties encountered in the forward translation of scientific concepts developed in currently used standard rodent EAE into effective treatments for patients shows that pathogenic mechanisms in these models inadequately replicate the situation in the patient. As discussed elsewhere,^44^ much may be learned from a reverse translation analysis of the reasons why a promising treatment fails in the clinic. With such information in hand the relevance of the preclinical models can be improved.

2. Replacement: When two models score equally on the Relevance criterion, the model in the lowest available species should always be used. As an example, the EAE initiation mechanism in marmosets essentially replicates the mouse EAE model,^8^ which has been useful for developing agents that show satisfactory effects in relapsing-remitting MS.^45^ So, when there are no barriers with respect to species specificity of a new drug there is no reason to use a primate disease model. However, the pathogenic mechanisms that drive conversion of RRMS to progressive MS are not recapitulated in the current mouse EAE models.^46^ None of the currently available treatments exerts a substantial clinical effect on secondary progressive disease. Also for the small subset of patients with primary progressive MS (10%) in which the disease is progressive from onset no effective treatment is available. This means that for the adequate treatment of progressive MS novel drugs need to be developed.

A pathological hallmark of progressive MS and the suspected cause of the accumulating irreversible neurological problems is neurodegeneration by mitochondrial dysfunction.^46^ One might therefore assume that the available neurodegeneration models, either established by genetic modification or induced with toxins, might deliver useful drugs for the treatment of progressive MS. However, this seems not to be the case. The possibility that progressive MS is driven by an unusual pathogenic mechanism that has not yet been modeled in experimental animals should be considered.^47^ The identification of progressive MS-like central nervous system (CNS) pathology in the atypical MOG34-56/IFA marmoset EAE model supports that possibility.

3. Refinement: Reduction of discomfort is essential, not only for the benefit of the animals in experiment, but also for the quality of the data. There are multiple sources of discomfort in the EAE model, each potentially having a different impact on individual animals. For example, NHP are social animals with a high level of consciousness. Moreover, the high social needs requires pair-housing of monkeys in experiment. Other sources of discomfort can less easily be removed, such as the loss of neurological functions that is inherent to the EAE model. One important achievement in the primate EAE model has been the replacement of CFA for the more animal-friendly IFA. However, as explained above, refinement of a model may increase the sensitivity to variation of environmental factors.

4. Reduction: Reduction of the variability that is inherent to primate autoimmune disease models can be achieved by using genetic selection markers for disease susceptibility. These have been defined for a rhesus monkey collagen-induced arthritis model of RA^48^ and a rhesus monkey EAE model induced with myelin basic protein in CFA.^49^ Such markers have not been found in the marmoset EAE model as both the EAE-initiation as well as the EAE progression mechanisms are regulated by invariant MHC molecules.^8^ An important advantage of the marmoset model, however, is that immunologically alike twins can be used.^9^ By using such chimeric twins in a paired two leg study design relevant information can be obtained from relatively small-sized study groups.

An investment can also be made in procedures with which more information can be obtained from individual animals in an experiment, such as via imaging or biomarker analysis in excretory fluids. For the marmoset EAE model, nuclear magnetic resonance-based imaging methods have been implemented with which the evolution of brain pathology could be longitudinally monitored independent of clinical scores.^19^ Also the use of sensitive behavioral tests can help to detect subtle motoric defects, which are not visible at inspection of animals in the home cage.^50^

## Concluding remarks

Preclinical research with NHP is challenging due to the high costs, while funding is tight. Scientists working with these precious models are also facing increasing problems with ethical restrictions and public opposition. NHP nevertheless provide essential and translationally relevant disease models, which may be an appropriate answer to the pressing need of animal models that better predict clinical success of a new treatment in patients. The central theme of this publication is the conflict between clinical relevance and statistical significance. Reviewers of projects and publications clearly appreciate low *p*-values. However, in the more than 25 years research experience in this field I never received a satisfactory answer to the question why a scientific finding in ten 10–12 weeks old mice from a single inbred/SPF mouse strain is better news for the genetically heterogeneous late-adult MS patient population than a finding in 5 late-adult monkeys from a genetically heterogeneous colony. Is this the *p*-value addiction that Peter Bacchetti argued against? I have often objected against the pressure to increase test group size in primate EAE models with the only purpose to obtain a powered therapy test, while it seems acceptable to show a clinical effect in a standard EAE model in a single mouse model with a poor translation record. As argued by Bacchetti *et al.*,^43^ also in my own experience large studies were rarely more informative than small studies, while the cumulative discomfort of the experiment as well as the costs increased proportionally with the group size. Only when a new therapy is partially effective, such as in our marmoset EAE study with the anti-CD127 mAb,^39^ a larger study can reveal whether a beneficial effect concerns more than a few animals.

I posit that reviewers of projects and editors of scientific journals do justice to the special nature of non-human primate models of human disease and free themselves from the traditional sample size dogmas. Heterogeneity in the incidence and course of a disease and the response to treatment is inherent to a model that is exposed to variable influence of genes and environment. Finally, investment in the clinical relevance and refinement of a disease model may inevitably lead to enhanced sensitivity to external factors.
